# Monitoring Land Degradation Dynamics to Support Landscape Restoration Actions in Remote Areas of the Mediterranean Basin (Murcia Region, Spain)

**DOI:** 10.3390/s23062947

**Published:** 2023-03-08

**Authors:** Marzia Gabriele, Raffaella Brumana

**Affiliations:** Politecnico di Milano, Department of Architecture, Built Environment and Construction Engineering (ABClab-GICARUS), Via Ponzio, 31, 20133 Milan, Italy; raffaella.brumana@polimi.it

**Keywords:** land degradation, landscape restoration, Google Earth Engine, desertification, NDVI, R script, Sentinel-2, MODIS, rGEE, cloud computing

## Abstract

This study aims to develop a workflow methodology for collecting substantial amounts of Earth Observation data to investigate the effectiveness of landscape restoration actions and support the implementation of the Above Ground Carbon Capture indicator of the Ecosystem Restoration Camps (ERC) Soil Framework. To achieve this objective, the study will utilize the Google Earth Engine API within R (rGEE) to monitor the Normalized Difference Vegetation Index (NDVI). The results of this study will provide a common scalable reference for ERC camps globally, with a specific focus on Camp Altiplano, the first European ERC located in Murcia, Southern Spain. The coding workflow has effectively acquired almost 12 TB of data for analyzing MODIS/006/MOD13Q1 NDVI over a 20-year span. Additionally, the average retrieval of image collections has yielded 120 GB of data for the COPERNICUS/S2_SR 2017 vegetation growing season and 350 GB of data for the COPERNICUS/S2_SR 2022 vegetation winter season. Based on these results, it is reasonable to asseverate that cloud computing platforms like GEE will enable the monitoring and documentation of regenerative techniques to achieve unprecedented levels. The findings will be shared on a predictive platform called Restor, which will contribute to the development of a global ecosystem restoration model.

## 1. Introduction

The term “desertification,” as it is defined in the latest *World Atlas of Desertification* (WAD3), is considered to be “a nebulous, all-encompassing concept” [1]. This is mainly due to the complex nature of the phenomenon, which makes it difficult to measure its main causes and consequences and to agree on a standard methodology to assess the multiple factors that contribute to it. This statement confirms the high level of complexity of land degradation (LD) globally and draws attention to the various ecological and socio-economic threats it poses, including its multidimensional nature, causes, spatial footprint, and multiple consequences for both ecological and anthropogenic systems worldwide, as well as the global climate system [2]. In the current scenario, the intensification of LD (the primary environmental disturbance found in dryland systems) is expected to increase, along with the anthropic crisis associated with rising poverty, food insecurity, human migration, and regional political instability [3]. The intrinsic correlations between socio-economic and ecological dynamics connote land degradation as the irreversible condition resulting from stages leading to ecological losses in terms of complex soil properties, leading to land impoverishment and land abandonment. In 2007, the United Nations Convention to Combat Desertification (UNCCD) COP 8 [4], approved the first ten-year strategic plan and framework for implementing the UNCCD Convention. The plan underlined the Convention implementation as a mean of preventing, controlling, and reversing desertification/land degradation, contributing to the reduction of poverty, and promoting Sustainable Development Goal 15-Life on Land [5]. The plan emphasized that combating drought, land degradation, and desertification was an international priority. “Combating desertification/land degradation” then meant targeting all stages of the process of land degradation in drylands, even if the state of desertification had already been reached. Further developments took place during COP 21 [6] of the UN Framework Convention on Climate Change (UNFCCC) in Paris in 2015 when the UNCCD country parties reached a breakthrough agreement on the Land Degradation Neutrality (LDN) [7] concept. The LDN concept aims to encourage implementing an optimal mix of measures designed to avoid, reduce, and/or reverse land degradation to achieve a state of no net loss of healthy and productive land. During the UNCCD COP 13 [8] Convention that took place in September 2017 in Ordos, China, countries agreed on a new global roadmap to address land degradation: the UNCCD 2018–2030 Strategic Framework. This is still the most comprehensive global commitment to achieving land degradation neutrality (LDN), restoring the productivity of vast swathes of degraded land, improving the livelihoods of over 1.3 billion people, and reducing the impacts of drought on vulnerable populations. To contextualize this research within the LDN framework and the aims of the Ecosystem Restoration Camps [9] world project, it is important to note that among the various actions listed to achieve LDN principles, efforts to reverse land degradation through restoration or rehabilitation of degraded land are crucial assets for promoting sustainable land management (SLM) practices. This approach can help to avoid or reduce degradation and aligns with the response hierarchy of avoid > reduce > reverse land degradation, which articulates the priorities for planning LDN interventions [7].

## 2. Stepping into a New Decade of Monitoring Ecosystem Restoration Camps

The fifteenth session of the Conference of the Parties UNCCD COP 15 [10] of the United Nations Convention to Combat Desertification (UNCCD) was held in Abidjan, Côte d’Ivoire, from 9 May to 20 May 2022. The conference theme was “Land. Life. Legacy: From scarcity to prosperity,” and its primary goal was to call for action to ensure the sustainability of land and life on this planet in pursuit of Sustainable Development Goal 15 (Life on Land) [5]. This effort seeks to benefit both present and future generations.

The Ecosystem Restoration Camps (ERC) Foundation is an international movement that aims to restore degraded lands around the world through the establishment of local living labs, where volunteers work together to implement landscape restoration strategies. Currently, ha. under restoration number 8.960, and the trees and plants planted are 2.713.296 [9]. As the ERC movement continues to grow and expand, it is essential to have a monitoring framework in place to track the progress of restoration actions, assess the effectiveness of different strategies, and share knowledge among the various camps. This can be particularly challenging in areas where there is limited access to resources, including technology, transportation, and infrastructure. In fact, the conditions of the areas under restoration must be referred to as isolated communities, which rely on simple yet efficient methodologies and models to track changes in the restoration actions monitoring and management. Therefore, it is essential to use methods that are practical and appropriate to the local context while still providing accurate and useful information about the restoration process. This can help ensure that the restoration efforts are successful and sustainable over the long term.

The methodology presented in this study is the first step in setting up a common reference framework for the ERC Foundation, which will be scalable to other camps around the world. The study proposes a time-efficient approach to the methodological structurization of Indicator 16—Above Ground Carbon Capture (referring to the Soil Framework [11]) by direct estimation of above-ground carbon stocks based on satellite-derived reflectance data. This approach can be applied across multiple camps and landscapes, allowing for consistent monitoring and comparison of restoration efforts.

As we step into the UN Decade on Ecosystem Restoration, it is crucial to have a standardized monitoring framework in place for restoration initiatives like the ERC movement. This will help to ensure that restoration efforts are effective and sustainable and contribute to the larger goal of combatting land degradation, climate change, and biodiversity loss. The establishment of a common reference framework for ERCs will enable the movement to leverage data and knowledge from different camps and landscapes and facilitate the sharing of best practices and strategies for effective ecosystem restoration.

### 2.1. A New Envisioning for the Indicator 16—Above Ground Carbon Capture

The data needed to foster Indicator 16 will be extracted by analyzing trend values of ongoing restoration actions using remote sensing techniques. This will involve integrating vegetation indices such as the Normalized Difference Vegetation Index (NDVI) with the help of Google Earth Engine’s (GEE) powerful cloud computing technologies. The GEE API will be called from within R (rGEE) [12] to structure a coding workflow that improves the temporal and spatial resolution and accuracy of the data. By doing so, it will be possible to monitor landscape regeneration and land restoration actions without needing to download data from the satellite. The next section will focus on recent works that have utilized GEE for environmental monitoring. This discussion will cover innovations in literature, cutting-edge applications, and approaches that make use of GEE. Cloud-processing systems like GEE, which offer free access to Earth Observation (EO) datasets worldwide [13], are a promising development for dynamic environmental monitoring. GEE has become a provider of solutions to the main bottlenecks encountered in processing satellite Big Data for environmental applications in the field of Earth Observation studies [14,15]. The need to manage large amounts of data efficiently for monitoring purposes is one such bottleneck. GEE is flexible and enables more researchers, especially those in developing countries with limited resources, to access and analyze data. Integrating GEE technology in scientific research focused on land monitoring in the EO domain could foster innovative potentials to simulate hazards and pressures to identify mitigation solutions that support real-time responders as well as medium- to long-term active solutions.

### 2.2. Google Earth Engine Applied to Land Monitoring Framework

The utilization of GEE technology and NDVI indicators has become a prevalent approach in environmental monitoring for remote and challenging regions of the planet. In a recent study, GEE was used to capture logging and burned area mapping (abiotic and biotic disturbances) across a peri-urban forest and their effects on spatiotemporal changes to erosion dynamics [16]. Moreover, another researcher developed a GEE code to estimate the properties of vegetation phenology in fire-affected areas [17]. In [18] GEE was used to evaluate the performance of automatic detection of flood-inundated areas in Charikar city, Parwan province, Afghanistan, by using the spectral index technique based on the relative difference in the Normalized Difference Vegetation Index (rdNDVI) between pre- and post-event Sentinel-2 images; in [19] GEE was used in combination with R (rGEE), R coding workflow, bridging the gap between R and the multi-petabyte catalogue of remotely sensed data available in Google Earth Engine (GEE) to study Normalized Difference Vegetation Index information from the MOD13Q1 data product for 12.344 GPS animal location in Central Africa; in [20] the GEE analysis was performed using JavaScript commands in Google Earth Engine, to study the impact of the Syrian crisis on agricultural land abandonment by evaluating phenological characteristics of NDVI and NDMI during the crisis (2013–2021), compared to the phenological profiles for the period before the crisis (1986–2010; in Zambezia province of Mozambique, GEE was used to create an assessment methodology leveraging the power of open remote sensing data and tools to map smallholder and large-scale cropland dynamics, integrating categorical and continuous training and validation data obtained from field surveys [21]. Concerning the application of GEE in the Land Degradation and desertification evaluation, the latest study in the field suggests approaching a more dynamic evolution, as [22] which proposed a hybrid development of the MEDALUS framework in the Google Earth Engine (GEE) environment, applied to the Blue Nile Basin study area degradation (MEDALUS-GEE framework). The study mapping and assessment of the Quality indexes from the ESA framework were fully implemented in the GEE environment. In this direction, the Land Degradation Monitoring Project (LDMP), a project funded by the Global Environment Facility (GEF), designed a free and open-source tool—Trends.Earth [23]. The biggest Trends.Earth feature is in the use of cloud computing—by using Google Earth Engine (GEE), thus, making it possible for users with limited computing capacity and without expert knowledge of cloud computing to perform Vegetation Index (VI) calculations to evaluate Land Degradation on large datasets at large scale areas (regions).

## 3. Materials and Methods

### 3.1. Land Restoration Actions Monitoring—The Area of Interest

**Figure 1:**
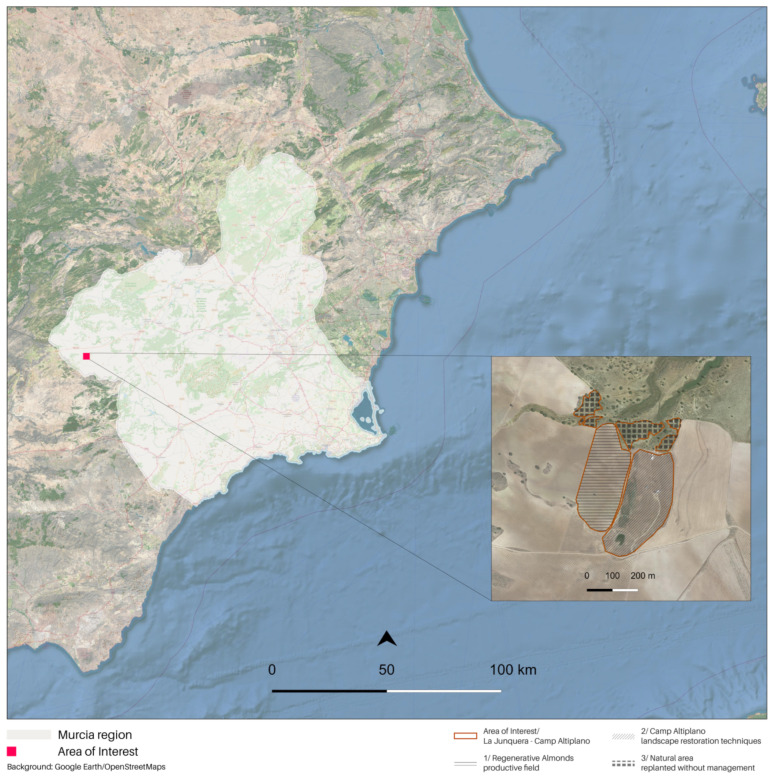
The study area of interest, framed in the Region de Murcia, at the top right: the area of interest specificities: ERC Camp Altiplano, where the landscape regeneration techniques are being implemented, and its proximity surroundings, the regenerative almonds on the left and, in the upper portion, the natural area.

The focus of this study is Camp Altiplano, a pioneer in the Ecosystem Restoration Camps (ERC) network. It is located in the Region de Murcia, Spain, and was established in April 2017. ERC is a non-profit foundation that aims to promote local community engagement, research, and training to support ecosystem restoration. Camp Altiplano is situated within La Junquera, which is a small settlement and farm and covers an area of 5 hectares. The research area encompasses the entire La Junquera-Camp Altiplano area, including both restoration and regenerative actions (Figure 1). The site is located at an elevation of 1.100 m and belongs to a high-steppe ecosystem with a semi-arid climate and an annual rainfall of 250 mm. These geological and extreme climatic conditions have worsened the anthropological pressure on the area, including deforestation, industrial agriculture, and water resources exploitation, making restoration actions challenging. The ERC Camp Altiplano Restoration Plan has invested in experimental landscape regeneration techniques. Some examples of soil management practices that can improve water infiltration and reduce erosion and evaporation include the use of swales for water management, deep ripping to de-compact topsoil layers, and planting cover crops to enrich the soil with biomass. These techniques have led to an increase in water, which has been used to grow vegetation and perform nature-based soil works. The overall holistic management of the area promotes soil microbiology and self-regulating systems capable of building soil nutrients. The research focuses on the pioneering ERC Camp Altiplano experimental landscape restoration techniques that have been specifically designed to adapt to the semi-arid climate, characterized by dry summers, heavy autumn rains, and winter lows of −12 degrees.

The main objective of broadening the methodological investigation to the entire area was to compare the resilience of different ecosystems over the long term. By examining the productive almond tree area adjacent to Camp Altiplano in La Junquera, the tangible differences between the camp’s approach and the nearby management of almonds using new regenerative agricultural techniques aimed at more sustainable production and soil regeneration were revealed. Additionally, another area was examined for comparison purposes: a natural area located above the camp that has been replanted and regenerated using a completely different approach. The primary management of this area is left to nature without any human interference or secondary regulation.

-Earth Observation (EO) monitoring will identify the hotspots where restoration actions are being performed and measure their impact on net primary production. This will help to understand the differences resulting from the various techniques being tested at Camp Altiplano. By comparing the results with the overall landscape regeneration in the vicinity, the tangible impacts of the in-place restoration can be outlined, leading to a comprehensive understanding of the achieved ecosystem resilience over time. Collecting feedback and return outputs of the indicators contained in the Soil Framework will help to create a reference framework for restoration techniques, allowing for the future scalability of the most successful and impactful techniques in other ERC foundation areas.

The forthcoming Materials and Methods section is organized into three sub-sections (Section 3.1.1, Section 3.1.2, Section 3.1.3) to introduce regeneration and restoration techniques and describe the current actions taking place in the area of interest, La Junquera-Camp Altiplano. Sub-Section 3.2 will focus on the technical aspects, providing a detailed description of the three parts of the rGEE code and the selected Vegetation Indices, specifically the Normalized Difference Vegetation Index (NDVI). This includes sub-sections on Data Collection and Methodology, as well as descriptions of the first (MODIS/Terra-MOD13Q1 NDVI), second (COPERNICUS/S2_S), and third (sample COPERNICUS/S2_SR SOS-EOS and WS NDVI) parts of the rGEE code. The section is supplemented by tables containing metadata from both MODIS/Terra-MOD13Q1 NDVI and COPERNICUS/S2_SR GEE repository, as well as tables georeferencing the area of interest and the polygons of the sampled areas in the projected CRS WGS 84/UTM zone 30N.

#### 3.1.1. La Junquera—Regenerative Almonds, Productive Field 

**Figure 2:**
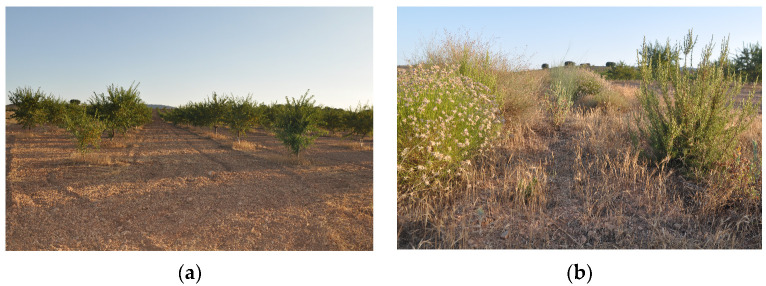
(**a**) Keylines, regenerative almonds; (**b**) aromatics in the study area site, ERC Camp Altiplano—author’s photo 07/2022.

La Junquera farm has planted regenerative almond trees with the aim of achieving profitable almond production using regenerative agricultural techniques and the keyline design developed by P. A. Yeomans in Australia [24]. The keyline is a contour line that follows the natural shape of the land, going through the keypoint where the valley becomes steeper. Cultivation follows the same patterns above and below the keyline to redirect water and prevent erosion. This helps achieve zero run-offs and holds all water in the soil and biosphere (Figure 2). The almond trees are planted in rows based on regenerative agriculture principles and include varieties such as guara, advisor, and antoñeta, interspersed with Russian olive, black locust, and native fixing shrubs. A distance of 7 m is kept between each row of trees, where three rows of mixed aromatics and native perennials are planted to allow for feasible almond harvesting with a tractor. The remaining space is dedicated to a cover crop of barley, bitter vetch, and mixed native flowering plants.

#### 3.1.2. Camp Altiplano—Landscape Restoration Technique, Riparian Zone and Forest Garden

**Figure 3:**
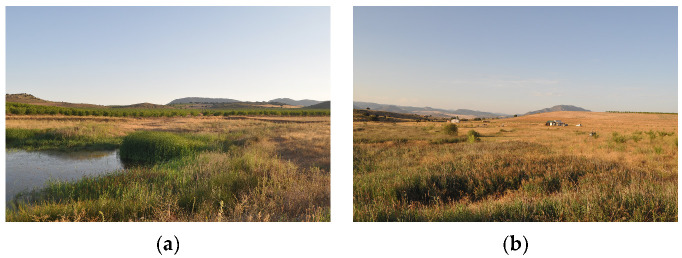
(**a**) Riparian zone and (**b**) forest garden, ERC Camp Altiplano—author’s photo 07/2022.

The initial step in restoring the ecosystem focused on the riparian zone near the man-made ponds at the camp (Figure 3). The western border of the camp has experienced water scarcity, making it an ideal location for ecosystem restoration. In 2017, three ponds were excavated primarily to retain water, thereby reducing topsoil nutrient loss due to heavy rain. The restoration of this area is crucial as the chosen species can provide food, shelter, and a habitat for wildlife, while also creating biomass and stabilizing the soil. Additionally, the vegetation provides protection against the strong south-west winds that can reach speeds of up to 90 km/h. The ponds serve as a water source for wildlife and act as a breeding ground for local fauna such as frogs, insects, and water snakes.

The forest garden, which covers 0.65 hectares of the camp’s surface area, is situated close to the riparian zone. Since 2018, restoration actions have been carried out in a trial-and-error approach, beginning with the planting of fruit, nut, and nitrogen-fixing trees with supportive guilds of plants in a 0.16-hectare area that partially covers the vegetable patch. The current planting season (2022–2023) involves expanding the forest garden upslope, filling spaces between swales with a mixture of perennial and annual vegetables underneath the developing trees, and establishing fruit trees, mixed aromatics, and perennials to attract bees and promote biodiversity.

#### 3.1.3. Camp Altiplano—Landscape Restoration Technique, Swales, and Natural Corridors

**Figure 4:**
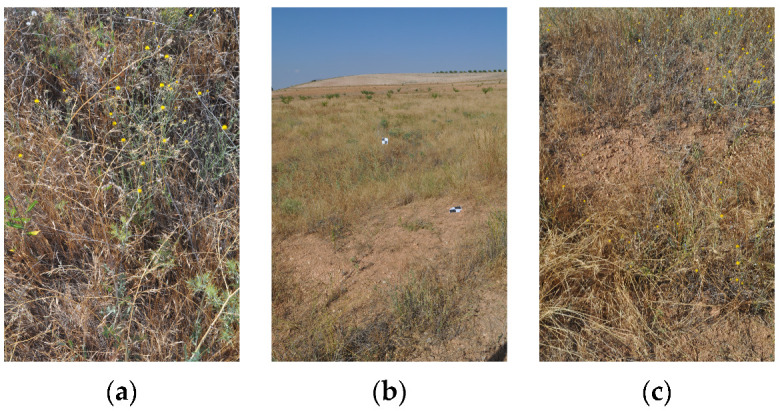
(**a**–**c**) Swales and natural corridors vegetation—author’s photo 07/2022.

During the summer of 2017, the Camp Altiplano team excavated swales that span the entire width of the plot. Swales are sunken trenches dug along contour lines that slow down water flow, allowing water to infiltrate the ground and horizontally feed the lands. This is especially important in arid climates where water conservation is essential. The first swale was planted in 2017 and continued through the autumn and winter of 2018. Swales were planted with native shrubs and trees to create natural corridors and attract birds and pollinators to the camp (Figure 4). Trees and larger shrubs were planted on the downslope of the swale, while aromatics were planted into the slope of the swale. The selected plants were chosen for their ability to thrive in a semi-arid environment and adapt to basic conditions with little care.

### 3.2. Data Collection and Methodology 

Timing plays a crucial role in the monitoring of restoration actions as it ensures consistent data collection, facilitating comparison and aggregation over time and across multiple ecosystem restoration projects worldwide. The Normalized Difference Vegetation Index (NDVI) [25,26] was selected as one of the indicators contributing to the methodological structurization of Indicator 16—Above Ground Carbon Capture [11]. It remains one of the most effective remotely sensed signals used to analyze vegetation activity in research. The NDVI is a simple numerical indicator that provides an estimate of vegetation health and monitors changes in vegetation and biomass over time. It is based on the fact that chlorophyll absorbs red light (RED), while leaves reflect near-infrared light (NIR). Due to vegetation pigment absorption (chlorophyll, protochlorophyllide), the reflected red energy decreases, while healthy leaves within the canopy scatter NIR energy strongly, resulting in increased reflected NIR energy [27,28,29,30,31]. The NDVI is computed as (1):

NDVI = (NIR-RED)/(NIR + RED).(1)

Over time, the annual integration of NDVI can serve as a substitute for estimating the distribution of annual biomass Net Primary Production (NPP) and fractional vegetation cover. As a result, NDVI has been utilized as an indicator for tracking land cover changes and desertification, as it is a useful proxy for vegetation productivity across a variety of biogeographic regions and spatial scales [32,33,34]. Temporal analysis of satellite-based NDVI is one of the major remote sensing tools which can identify the depletion of vegetation cover [35]. NDVI time series analysis can facilitate the examination of various ecosystem changes. For instance, integrated perspectives of photosynthetic activity can be obtained by analyzing annual mean or peak NDVI. Additionally, the detection of trends in NDVI time series can aid in the identification and quantification of recent alterations in ecosystem properties ranging from a local to global scale [36].

**Figure 5:**
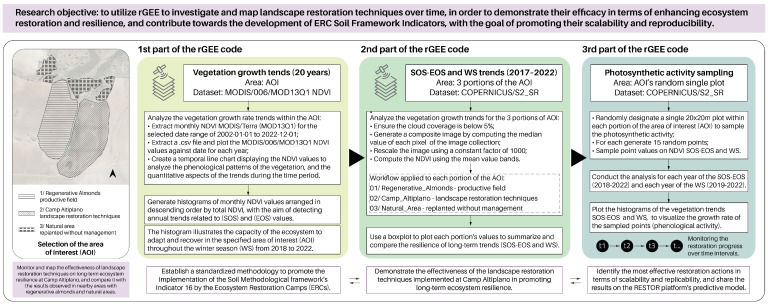
The methodological framework depicts the three primary components of the rGEE code workflow, along with the general research question and macro-objectives.

The methodological framework comprises three parts (Figure 5). The first part involved the utilization of rGEE code workflow to extract the MODIS/Terra-MOD13Q1 ‘NDVI’ Collection spatiotemporal phenological profiles of the monthly plotted NDVI values, with a 250 m spatial resolution, for a time interval of 20 years (2002–2022). This included the analysis of the NDVI phenological temporal line chart, which was further extracted and plotted in monthly histograms sorted by the total NDVI descending values [37,38]. The first part of the methodology was crucial for determining the growth rate, which was the highest registered amplitude in the NDVI profile. This enabled the subsequent setting of parameters for the second part of the workflow, which involved retrieving the COPERNICUS/S2_SR image collection (time interval: 2017–2022) to investigate the vegetation trends related to the start of the growing season (SOS) and end of the growing season (EOS) in the area of interest (AOI). The overall plotted phenological histogram derived from MOD13Q1 ‘NDVI’ clearly revealed that the restoration actions resulted in a resilient ecosystem capable of attaining a vegetation equilibrium during the Winter Season (WS). This finding was further explored in the second part of the methodology. The second part of the methodology involved utilizing rGEE code workflow to extract the COPERNICUS/S2_SR Image Collection and calculate NDVI, with a spatial resolution of 10 m, for each segment of the AOI, which included (1) Regenerative Almonds—productive field, (2) Camp Altiplano—Landscape restoration techniques, and (3) Natural area—replanted without management. This was conducted to enable yearly comparisons of the boxplot of NDVI values for SOS and EOS (April-May) and WS (December-March) for each segment of the AOI. The Third part of the methodology involved employing rGEE code workflow to extract COPERNICUS/S2_SR photosynthetic activity values for the SOS-EOS and WS time intervals to monitor and compare the specificities of the regeneration and restoration actions. This was accomplished by randomly selecting 15 points falling into defined 20 × 20 m plot constraints (time interval: 2017–2022).

#### 3.2.1. First Part of the rGEE Code—MODIS/Terra-MOD13Q1 NDVI

An important drawback of Google Earth Engine (GEE) is that it operates on an internet-accessible application programming interface (API) and a web-based interactive development environment (IDE), which can make it challenging to seamlessly integrate remote sensing products obtained from GEE directly into R [14]. The rGEE package [12] has overcome this limitation by allowing direct integration of the powerful computational infrastructure and multi-petabyte catalog of remote sensing products from GEE into R, enabling seamless data analysis workflows. The first part of the code workflow was created to extract monthly NDVI from the MODIS/Terra (MOD13Q1) version 6 product GEE Collection (Vegetation Indices 16-Day L3 Global 250 m) using polygonal geometric shapefile features defined within the constraints of the entire AOI. The analysis timespan covered uninterrupted data from 2002 to 2022. It is worth noting that the data values of the MOD13Q1 16-day composite do not represent an actual observation date for each pixel of the retrieved MODIS image.

The rGEE function ee$ImageCollection was used to retrieve a total of 479 images from the MODIS/006/MOD13Q1 ‘NDVI’ collection within the selected date range of 2002-01-01 to 2022-12-01. The ee_print() function was used to obtain information on the ImageCollection, including the overall metadata (Table 1), as well as exemplificative image (Table 2) and band (Table 3) metadata. The NDVI values were rescaled to a 0–1 range and renamed based on their date of acquisition using ee$Date(image$get(‘system:time_start’))$format(‘YYYY’). The function was applied to each retrieved image, and the resulting rescaled NDVI values were extracted from the camp POLYGON geometry using the ee_extract function and integrated with the shapefile containing the area geometry (AOI.shp) (Table 4) using the st_read function from the rGEE package. The resulting feature collection with the extracted pixel values was transformed back into an sf object using the ee_as_sf() function with the sf = FALSE argument. Finally, the sf_as_ee function transformed the resulting sf object into a GeoJSON format using geojson.json, which was then embedded in an HTTP request using the server-side objects (ee$Geometry$*).

#### 3.2.2. Second Part of the rGEE Code—COPERNICUS/S2_S

The second part of the code workflow aimed to extract the NDVI values between the SOS-EOS and WS time intervals from the COPERNICUS/S2_SR GEE Collection. The analysis covered a yearly period from 2017 to 2022 and applied a 5% cloud coverage threshold. The ImageCollection was filtered by month to obtain NDVI values for March, April, and May for the SOS-EOS interval and December, January, and February for the WS interval. The resulting monthly NDVI values were clipped for each portion of the AOI using the POLYGON Geometry filter bounds (Table 5, 01_Regenerative_Almonds; 02_Camp_Altiplano; 03_Natural_area). The ee_print() function provided information on an exemplar ImageCollection, such as its metadata, image metadata, and band metadata (Table 6, ImageCollection Metadata; Table 7, Image Metadata; Table 8, Band Metadata; shows the information contained in the 2017-time interval between the SOS-EOS NDVI, ‘2017-03-01′–‘2017-05-31′, Clipped on 01_Regenerative_Almonds) (Table 9, ImageCollection Metadata; Table 10, Image Metadata; Table 11, Band Metadata; shows the information contained in the 2021-time interval between the WS NDVI, ‘2021-12-01’–‘2022-03-01′ Clipped on 02_Camp_Altiplano).

To generate the composite image for each COPERNICUS/S2_SR yearly time interval of SOS-EOS and WS, the rGEE code employs the median composite function method. This method creates input images on a pixel-by-pixel basis by taking the median value (such as DN, TOA, or reflectance) from all cloud-free pixels in the image collection. This process preserves the phenology information and reduces data volume while producing an output image with high accuracy, similar to that of multi-temporal image data [39,40] (Table 12 and Table 13). The approach adopted in this work emphasizes the need for reduced storage space for two reasons. First, it allows for faster image analysis in the latter part of the methodology workflow. Second, it enables the creation of a remote data archive for use in areas with limited internet connectivity, a potential application with broad replicability in marginalized regions. The median composite image bands are rescaled using an image scale constant of 1000, and the NDVI is computed using the getNDVI function. This function extracts a normalized difference from the COPERNICUS/S2_SR “B8” and “B4” bands. The final NDVI product for both the SOS-EOS and WS time intervals, a 10-m spatial resolution image, is downloaded in Google Drive using the ee_Initialize(drive = TRUE) and ee_raster <- ee_as_raster functions, with a scale factor of 10 and crs = “EPSG:32630”.

#### 3.2.3. Third Part of the rGEE Code—Sample COPERNICUS/S2_SR SOS-EOS and WS NDVI 

**Figure 6:**
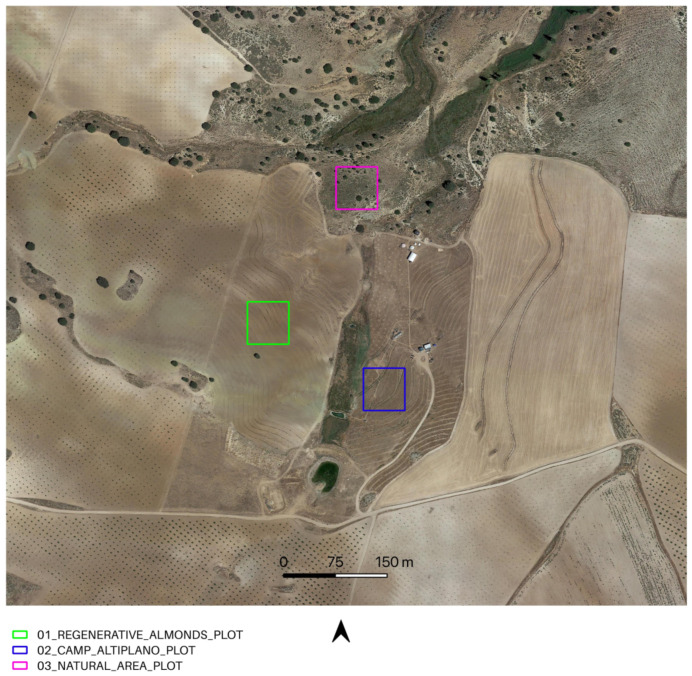
01_REGENERATIVE_ALMONDS_PLOT.shp samples an exemplificative portion of the regenerative agriculture almonds planted with the keyline approach; 02_CAMP_ALTIPLANO_PLOT.shp samples an exemplificative portion of the landscape restoration actions, in the specificity of the forest garden extent, encompassing a portion of the riparian vegetation; 03_NATURAL_AREA_PLOT.shp samples a portion in the upper AOI, reforested with landscape restoration techniques and left to natural management, without human interference.

In the third and final part of the methodology workflow, the Soil Framework Indicator 16—Above Ground Carbon Capture is completed by extracting NDVI values from 15 randomly selected points for a single plot chosen at random from each portion of the area of interest (AOI). This sampling approach allows for the evaluation of restoration and regeneration actions (as shown in Table 14 and Figure 6), as well as photosynthetic activity, during both the SOS-EOS and WS periods, for yearly time intervals t_1_, t_2,_ t_3_, … t_n_. The use of sampling to survey NDVI values is a well-established practice in Ecological science, as it enables the assessment of changes in the overall condition of a site or ecosystem relationships. Geometrically, the 15 sampling points are confined within a 20 × 20 m square polygon, with a mean distance of 15 m between points.

## 4. Results

The methodology using the rGEE code was effective and efficient in retrieving monthly NDVI values for twenty years (2002–2022) from the MODIS/006/MOD13Q1 ‘NDVI’ Collection and 10-m spatial resolution image rasters for six years (2017–2022) from the COPERNICUS/S2_SR Collection. This approach eliminated the need to download a large volume of satellite data to perform simple vegetation index computations during the time interval between SOS-EOS and WS. The use of rGEE facilitated the accessibility of remote sensing information by bridging R and Google Earth Engine, which allowed the utilization of all capabilities of the GEE geospatial tool. It is important to note that rGEE is not a native Earth Engine API like the Javascript or Python client. Instead, it involves the use of reticulate, an R package designed to enable seamless interoperability between R and Python. When an Earth Engine request is created in R, reticulate translates the request into Python and passes it to the Earth Engine Python API, which converts the request to a JSON format. Finally, the GEE Platform receives the request via a Web REST API, and the response follows the same path in reverse [12]. The majority of the transactions in GEE involve the preparation of a JSON object, which is then sent to the GEE server and translated into a final image or feature that can be viewed on a map or analyzed as properties using the getinfo() or ee_print() function. This methodology demonstrates that remote sensing information can be delivered dynamically to follow trends in land transformation, providing added value and opportunities for different operators to collaborate towards adaptive management actions. Although this research is still in its early stages, this approach has the potential to offer valuable insights and facilitate cooperation among different dryland stakeholders [41].

### 4.1. Analysis of the NDVI Phenological Profiles of the AOI—MODIS/006/MOD13Q1 NDVI (First Part of the rGEE Code)

**Figure 7:**
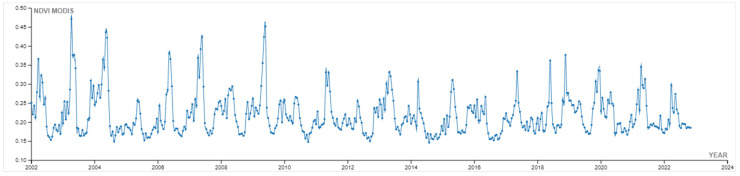
Spatiotemporal phenological profiles of plotted NDVI values from the MODIS/006/MOD13Q1 ‘NDVI’ Collection, clipped on the AOI.shp, time interval: 20 years (2002–2022).

**Figure 8:**
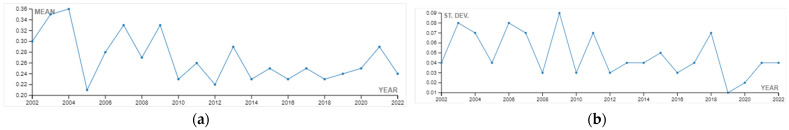
Yearly plotted NDVI values from the MODIS/006/MOD13Q1 ‘NDVI’ collection, clipped on the AOI.shp, time interval: 20 years (2002–2022); (**a**) yearly mean NDVI values; (**b**) yearly standard deviation NDVI values.

This paragraph describes the results obtained from the first part of the rGEE code, which involved retrieving and analyzing 20 years (2002–2022) of monthly NDVI values from the MODIS/006/MOD13Q1 NDVI collection. The NDVI data were plotted using the libraries “tidyverse” and “lubridate,” and for each year, the “ee_extract” function was used to retrieve the mean and standard deviation NDVI values. The resulting temporal line chart (Figure 7) visualizes the trends in vegetation phenology in the AOI over the 20-year period. The chart shows that the phenological profiles of the vegetation in the area are fluctuated and smooth, with a single peak, and the maximum average of the values registered during 2010–2017 is altered, possibly due to the long-term impact of intensive overgrazing, soil and water exploitation practices in the area. The chart also shows a slight increase in the peak growing season values from 2018–2021, which may be attributed to the positive effects of the ecosystem restoration actions taken in the area. However, the peak values in 2022 are lower, possibly due to extreme water scarcity and increased temperatures, which may have affected the overall survival rate of the 2021 planting season. The yearly extraction of mean and standard deviation values (Figure 8) allows for the interpretation of overall NDVI trends, which can be affected by agricultural practices, seasonal climate conditions, and restoration actions. The phenological profiles of the high steppe ecosystem semi-arid climate in the Camp Altiplano area are also clearly visible, with the peak growing season occurring in the time interval of March–May.

**Figure 9:**
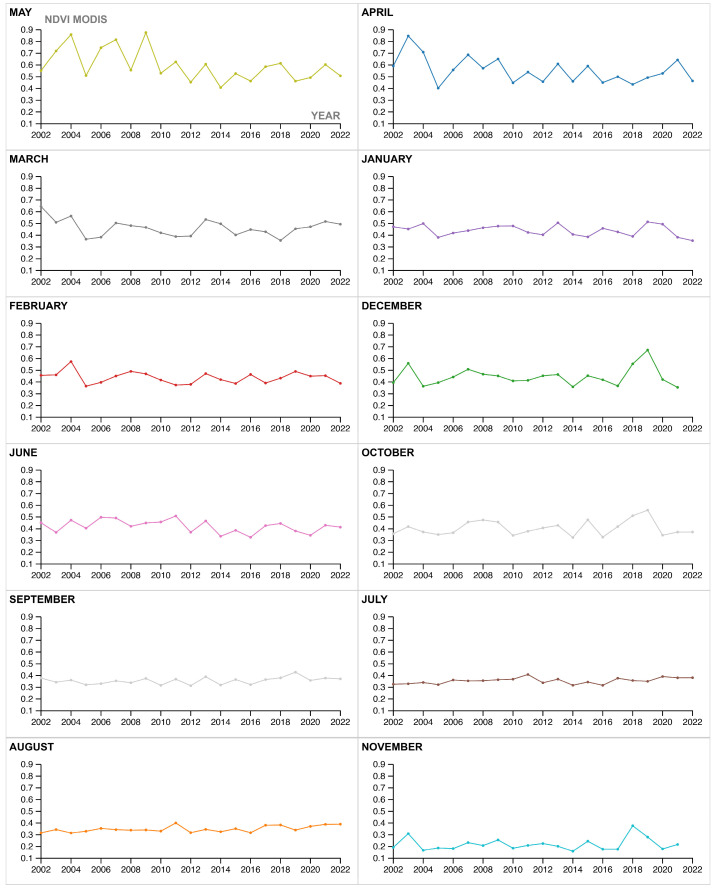
MODIS/006/MOD13Q1 NDVI vegetation trends monthly-phenological growth rate—the highest registered amplitude in the NDVI profile is registered at the start of the growing season (SOS) during March and April (germination) and the end of the growing season (EOS) in May (maturity). Moreover, it is to consider the interesting fact that the actions in place are contributing to the overall resilience of the ecosystem during the Winter Season (WS) months, from December to February.

Additionally, the start and end of the growing season, as well as the highest registered amplitude in the NDVI profile, are clearly evident in the extracted and plotted histogram monthly values sorted by total NDVI descending values (Figure 9). Specifically, the germination phase (SOS) occurs during March and April, while the maturity phase (EOS) occurs in May. Moreover, the graph emphasizes the vital achievement of the in-place methodology, which is to restore the active equilibrium of the vegetation throughout the year, including the winter season (WS). This is noteworthy since the winter season is typically challenging for ecosystems, and the restoration actions have contributed to increasing the ecosystem’s resilience during this time.

### 4.2. Analysis of the NDVI SOS-EOS and WS Time Intervals—COPERNICUS/S2_SR (Second part of the rGEE Code)

**Figure 10:**
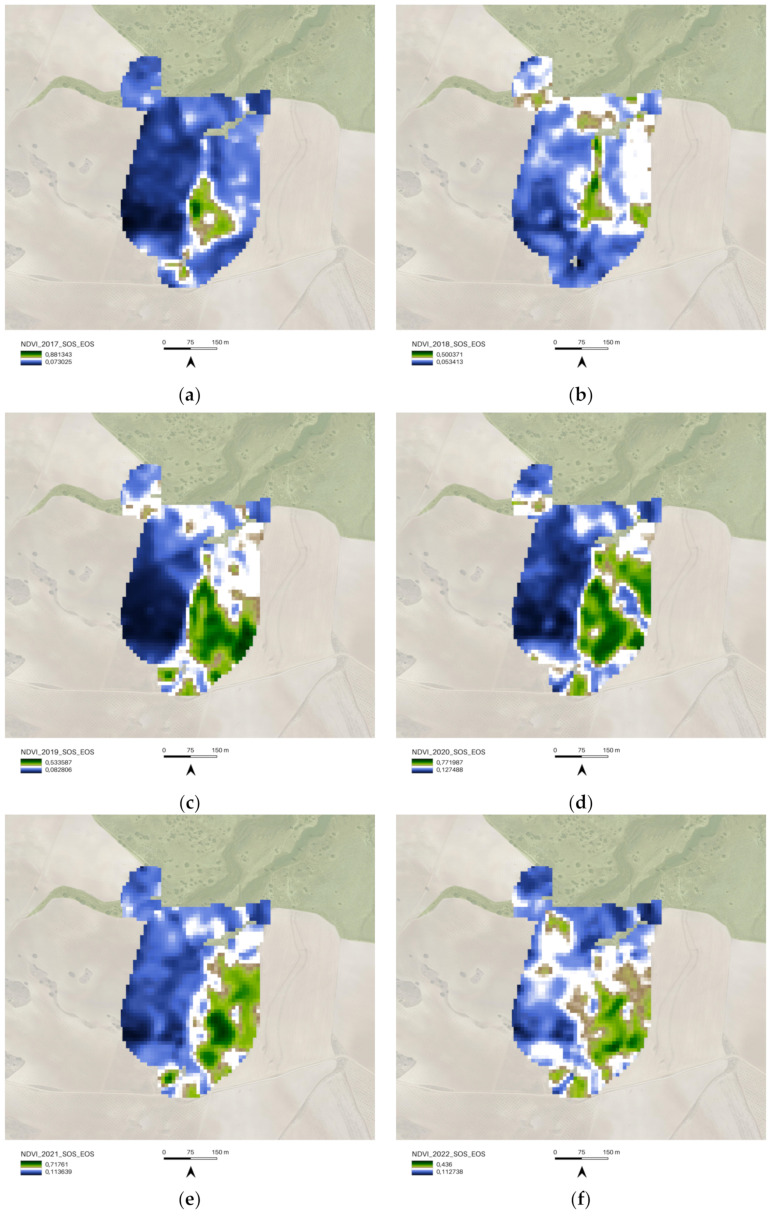
AOI.shp COPERNICUS/S2_SR NDVI vegetation trends SOS-EOS; (**a**) 2017 COPERNICUS/S2_SR filter dates SOS 2017-03-01, EOS 2017-05-31; (**b**) 2018 COPERNICUS/S2_SR filter dates SOS 2018-03-01, EOS 2018-05-31; (**c**) 2019 COPERNICUS/S2_SR filter dates SOS 2019-03-01, EOS 2019-05-31; (**d**) 2020 COPERNICUS/S2_SR filter dates SOS 2020-03-01, EOS 2020-05-31; (**e**) 2021 COPERNICUS/S2_SR filter dates SOS 2021-03-01, EOS 2021-05-31; (**f**) 2022 COPERNICUS/S2_SR filter dates SOS 2022-03-01, EOS 2022-05-31.

The preceding paragraph discussed the first part of the methodology, which involved using the MODIS/006/MOD13Q1 NDVI to analyze the entire area of interest (AOI) and determine the start of season (SOS), end of season (EOS), and winter season (WS) time intervals. The Second part of the methodology involved a more detailed analysis of different portions of the AOI with varying polygon boundaries, including 01_Regenerative_Almonds, 02_Camp_Altiplano, and 03_Natural_area. The investigation of the NDVI SOS-EOS in these three portions of the AOI began with a baseline in 2017, which marked the implementation of restoration and regeneration actions. The aim was to identify how the in-place actions were affecting biomass improvement in each area. It is important to note that weather conditions may affect agricultural status in a single growing season, and these factors were considered during data analysis. However, the focus of this research is on Indicator 16—Above Ground Carbon capture, and therefore, the results are based on the discussion of data extracted for the SOS-EOS and WS 10 m NDVI spatial resolution image (Figure 10). The spatial representation of information on the map enables the yearly monitoring of the area where land management practices perform better during SOS-EOS and WS.

**Figure 11:**
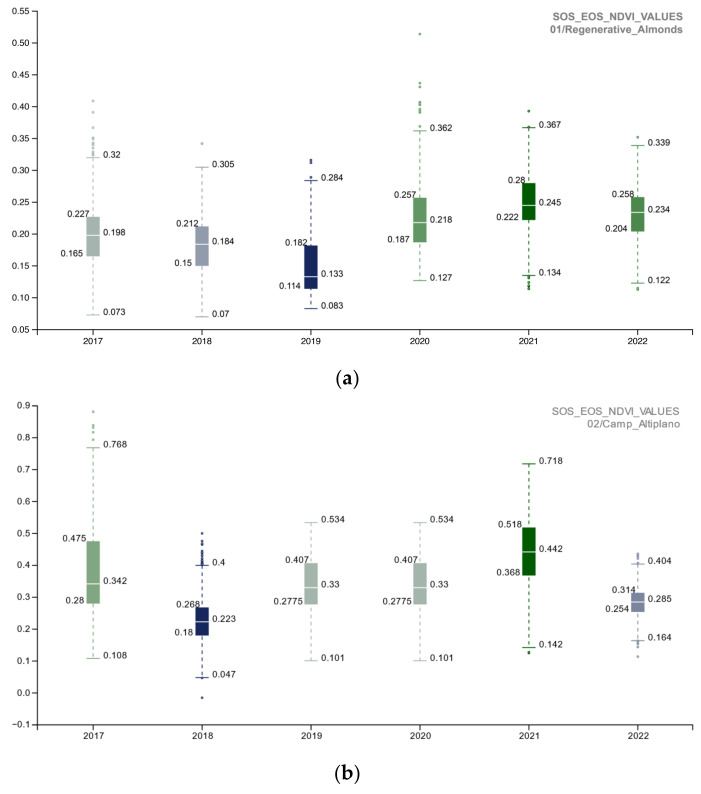

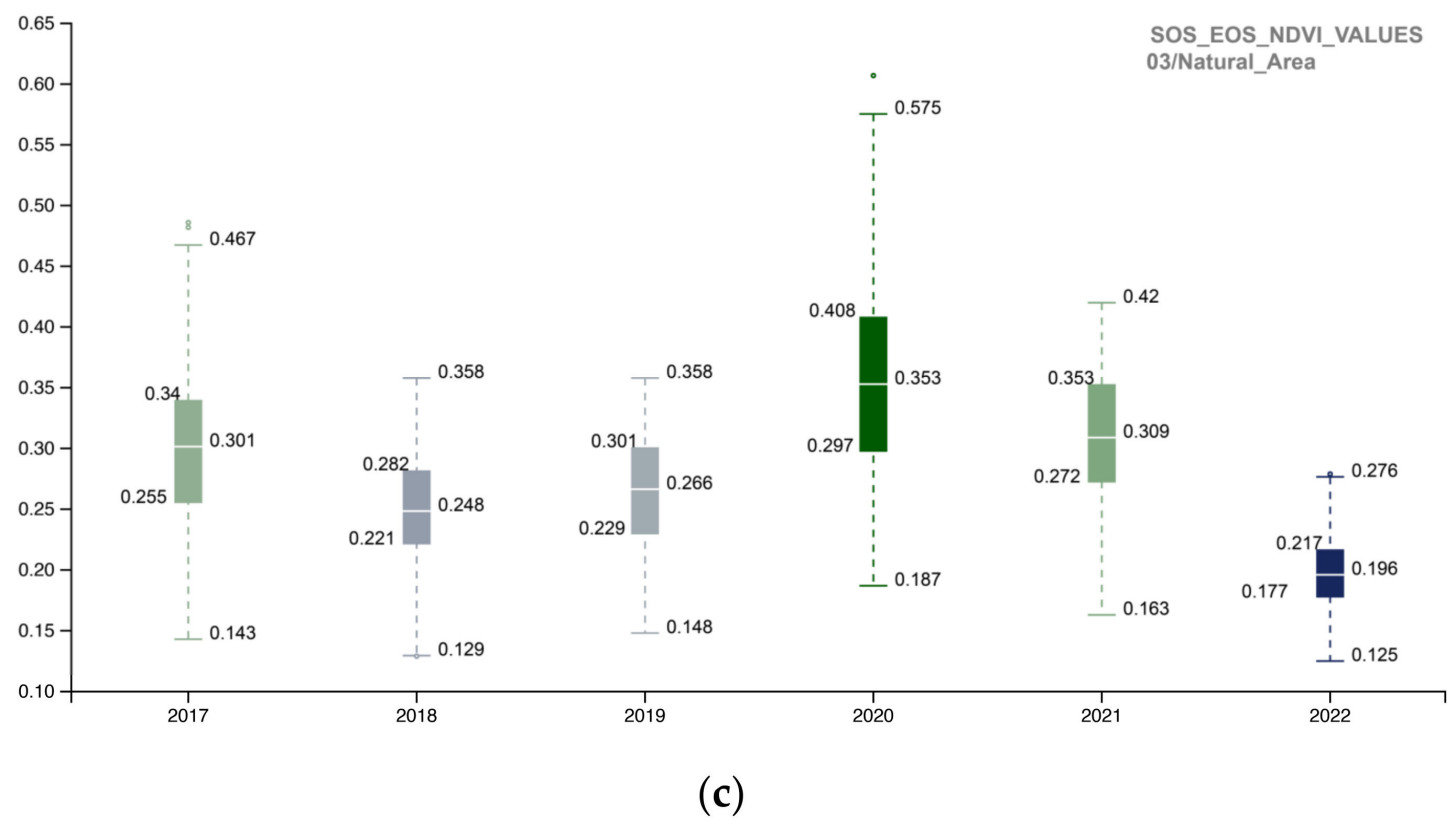
The boxplot summarizes and compares the NDVI SOS-EOS quantitative distribution for each portion of the AOI (**a**) 01_REGENERATIVE_ALMONDS.shp; (**b**) 02_CAMP_ALTIPLANO.shp; (**c**) 03_NATURAL_AREA.shp) with five standard statistics: the smallest value, lower quartile, median, upper quartile, and largest value; it is visible how the NDVI SOS-EOS 2017–2022 trends of the 02_CAMP_ALTIPLANO.shp area perform better overall in terms of being the most impactful virtuous restoration actions of the AOI (2019–2020); the 02_CAMP_ALTIPLANO.shp tends to be stable in time (resilience), with a slight decrease in 2022, maybe due to the poor survival rate of the trees and shrubs species planted the last years planting season (to be furthermore cross-related with other indicators of the Soil Reference framework).

**Figure 12:**
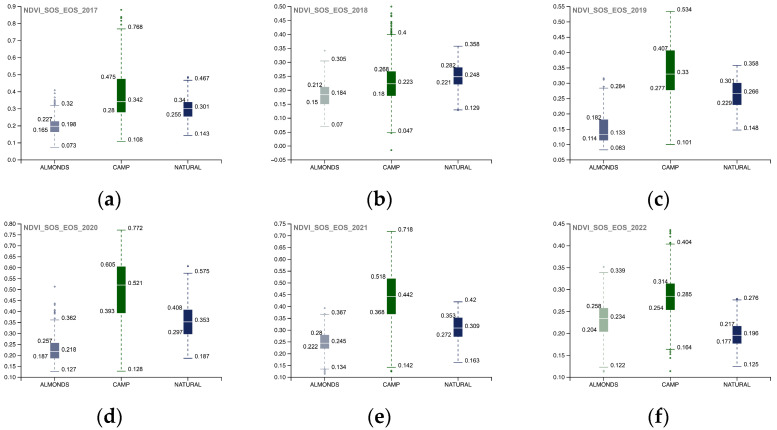
The yearly boxplots summarize NDVI SOS-EOS quantitative distribution values and compare each portion of the AOI (01_REGENERATIVE_ALMONDS.shp; 02_CAMP_ALTIPLANO.shp; 03_NATURAL_AREA.shp) with five standard statistics: the smallest value, lower quartile, median, upper quartile, and largest value. Here, the comparison allows for yearly analysis of the cumulated values of each portion of the AOI, specifically (**a**) 2017, (**b**) 2018, (**c**) 2019, (**d**) 2020, (**e**) 2021, (**f**) 2022. Landscape restoration actions of the Camp_Altiplano area stand out, tangibly proving the outputs of the ecosystem restoration methodologies.

To investigate the NDVI SOS-EOS values, a single-year boxplot comparison was completed for each portion of the AOI (Figure 11). The implemented methodology provided information about the landscape restoration actions’ outputs, and the comparison of the 02_Camp_Altiplano area with its surroundings, specifically the 01_Regenerative_Almonds and 03_Natural_Area (Figure 12), showed that in the long run, the most successful area was the one implementing the ERC Landscape restoration techniques. This indicates the effectiveness of these new methodological approaches (described in Section 3.1.2 and Section 3.1.3) in the field of ecosystem restoration.

**Inizio modulo**

**Figure 13:**
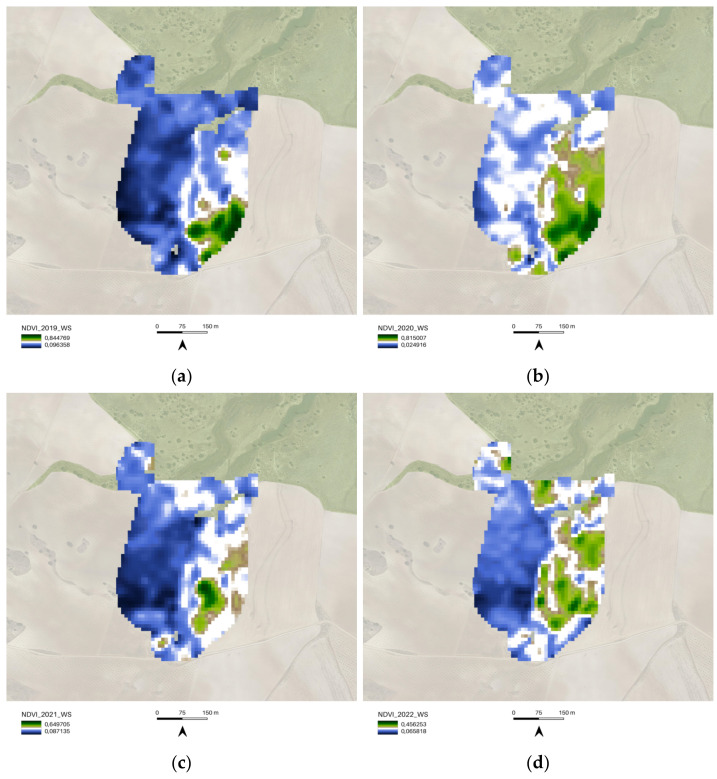
Camp_Altiplano_area.shp COPERNICUS/S2_SR NDVI; (**a**) 2019 COPERNICUS/S2_SR filter dates (YYYY-MM-DD) WS start 2018-12-01, WS end 2019-03-01; (**b**) 2020 COPERNICUS/S2_SR filter dates (YYYY-MM-DD) WS start 2019-12-01, WS end 2020-03-01; **(c**) 2021 COPERNICUS/S2_SR filter dates (YYYY-MM-DD) WS start 2020-12-01, WS end 2021-03-01; (**d**) 2022 COPERNICUS/S2_SR filter dates (YYYY-MM-DD) WS start 2021-12-01, WS end 2022-03-01.

**Figure 14:**
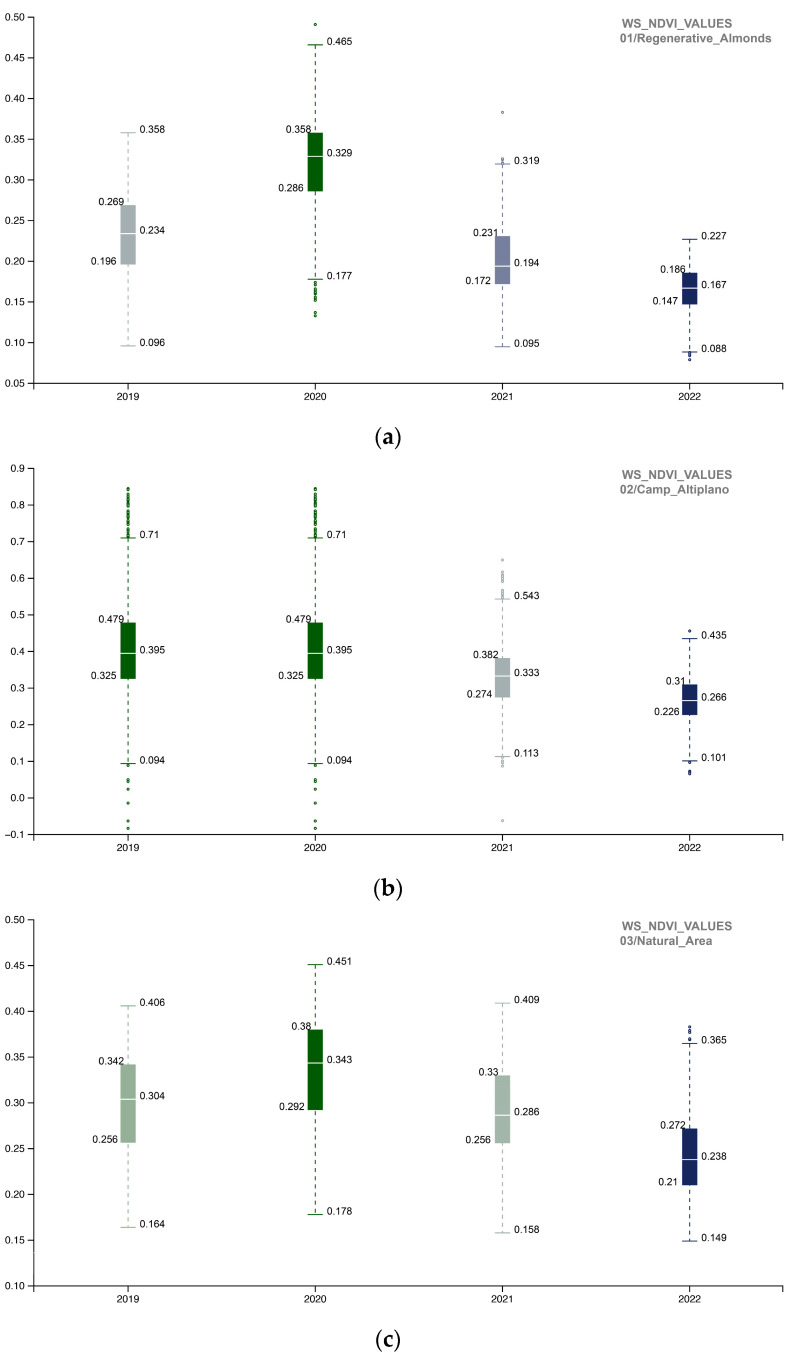
The boxplot summarizes NDVI WS quantitative distribution for each portion of the AOI (**a**) 01_REGENERATIVE_ALMONDS.shp; (**b**) 02_CAMP_ALTIPLANO.shp; (**c**) 03_NATURAL_AREA.shp) with five standard statistics: the smallest value, lower quartile, median, upper quartile, and largest value. It is visible that the 02_CAMP_ALTIPLANO.shp area reaches an overall resistance and stability (resilience) after the most impactful virtuous restoration actions (2019–2020) (to be furthermore cross-related with other indicators of the Soil Reference framework).

The following images depict and examine the results obtained from the WS time interval analysis. The WS analysis was conducted for the period spanning from 2019 to 2022 using a 10 m NDVI spatial resolution image (Figure 13). The spatialized NDVI map illustrates how the ecosystem restoration actions carried out in the 02_Camp_Altiplano area remain slightly active during the winter season, showing endurance and resilience throughout the year. The NDVI WS values were evaluated by comparing the single-year boxplot for each portion of the AOI (Figure 14), revealing that the 02_Camp_Altiplano area exhibits an overall trend of stability during the WS. The values for the year 2022 confirm the outputs of the SOS-EOS analysis, indicating a decrease in the quantitative distribution of the WS NDVI for the entire AOI area, which is probably related to climatic factors such as temperature and precipitation rather than specific restoration and regeneration actions. These elements need to be further analyzed within the Soil framework indicators.

**Figure 15:**
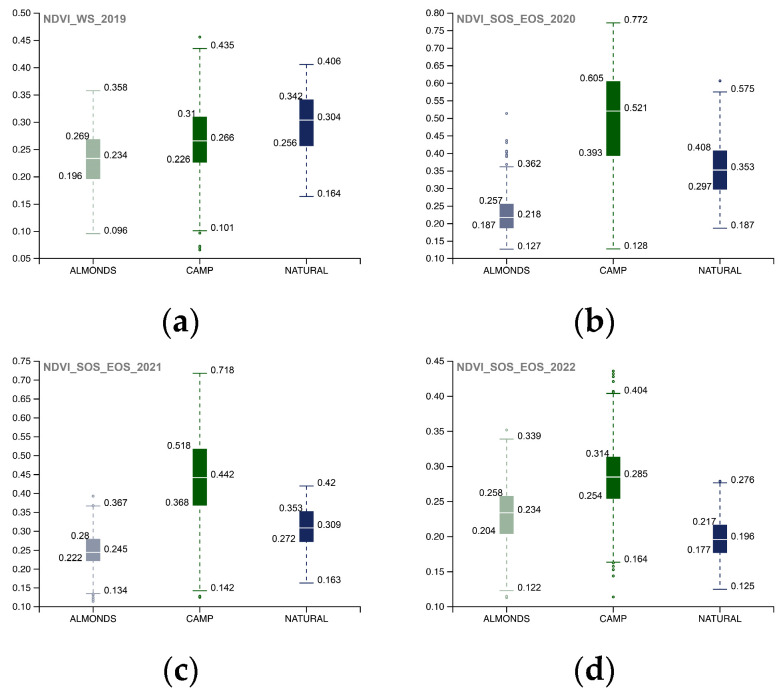
The yearly boxplots summarize NDVI WS quantitative distribution values and compare each portion of the AOI (01_REGENERATIVE_ALMONDS.shp; 02_CAMP_ALTIPLANO.shp; 03_NATURAL_AREA.shp) with five standard statistics: the smallest value, lower quartile, median, upper quartile, and largest value; here the comparison allows for yearly analysis of the cumulated values of each portion of the AOI, specifically (**a**) 2017, (**b**) 2018, (**c**) 2019, (**d**) 2020. Landscape restoration actions of the Camp_Altiplano achieve an overall coherent trend of vegetation activity during the winter period, tangibly proving the resilience of the ecosystem restoration methodologies.

Comparison of the 02_Camp_Altiplano area with its surrounding areas, 01_Regenerative_Almonds and 03_Natural_Area (Figure 15), allows for the yearly analysis of each portion of the AOI’s accumulated values, demonstrating that the resilience of the ecosystem restoration methodologies implemented in the Camp_Altiplano area enables the vegetation activity to remain stable even during the winter season.

### 4.3. Phenological Activity of the Plot Areas from the COPERNICUS/S2_SR NDVI (Third Part of the rGEE Code)

**Figure 16:**
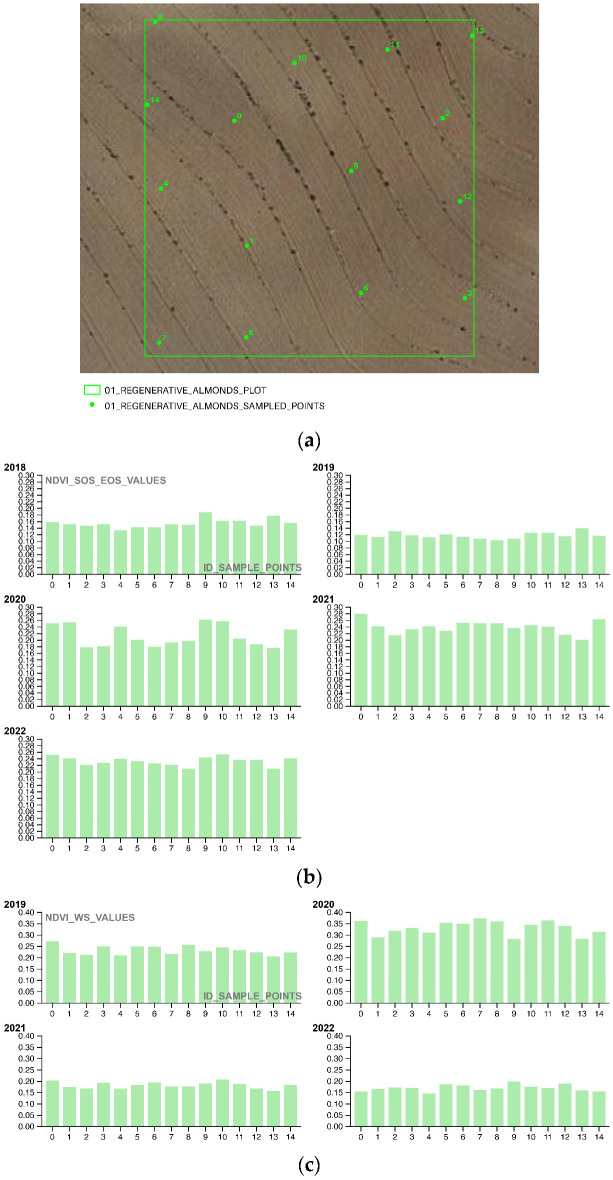
(**a**) The shapefile 01_REGENERATIVE_ALMONDS_PLOT.shp samples the COPERNICUS/S2_SR NDVI values of an exemplificative portion of the regenerative agriculture almonds planted with the keyline approach; (**b**) the COPERNICUS/S2_SR NDVI vegetation trends SOS-EOS phenological growth rate of the sample points (2018–2022) shows the trend in vegetation growth during the start of the growing season (SOS) and the end of the growing season (EOS) for the sampled regenerative agriculture almond plot; (**c**) the COPERNICUS/S2_SR NDVI vegetation trends WS phenological growth rate of the sample points (2018–2022) shows the trend in vegetation growth during the winter season (WS) for the sampled regenerative agriculture almond plot.

**Figure 17:**
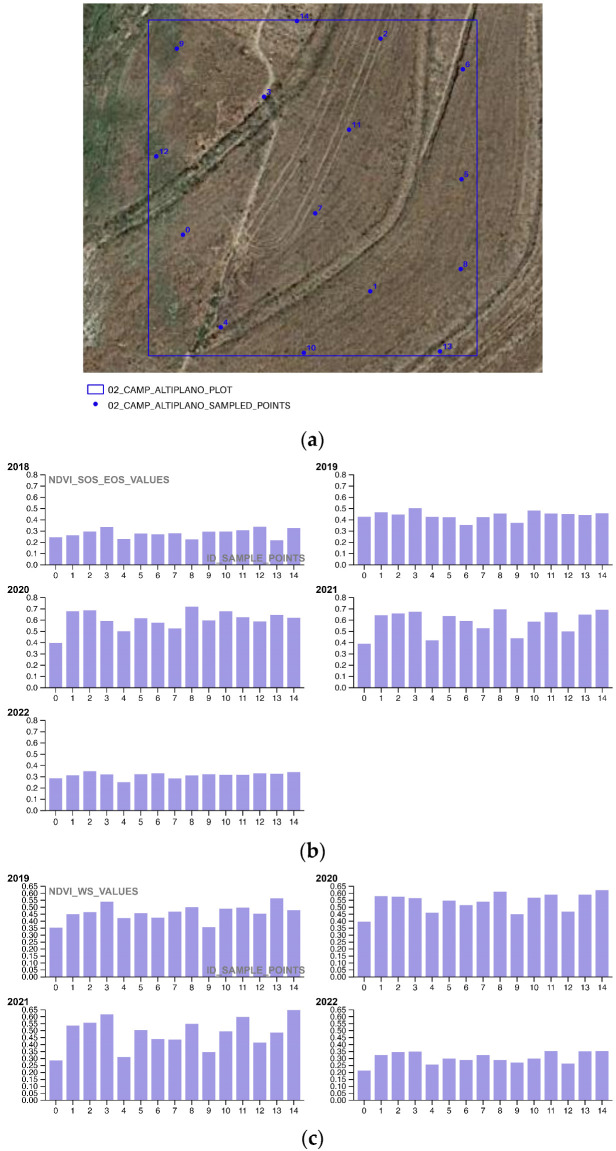
(**a**) The shapefile 02_CAMP_ALTIPLANO_PLOT.shp samples the COPERNICUS/S2_SR NDVI values of an exemplificative portion of the landscape restoration actions, in the specificity of the Camp Altiplano forest garden extent, encompassing a portion of the riparian vegetation; (**b**) the COPERNICUS/S2_SR NDVI vegetation trends SOS-EOS phenological growth rate of the sample points (2018–2022) shows the trend in vegetation growth during the start of the growing season (SOS) and the end of the growing season (EOS) for the sampled Camp Altiplano plot; (**c**) the COPERNICUS/S2_SR NDVI vegetation trends WS phenological growth rate of the sample points (2018–2022) shows the trend in vegetation growth during the winter season (WS) for the sampled Camp Altiplano plot.

**Figure 18:**
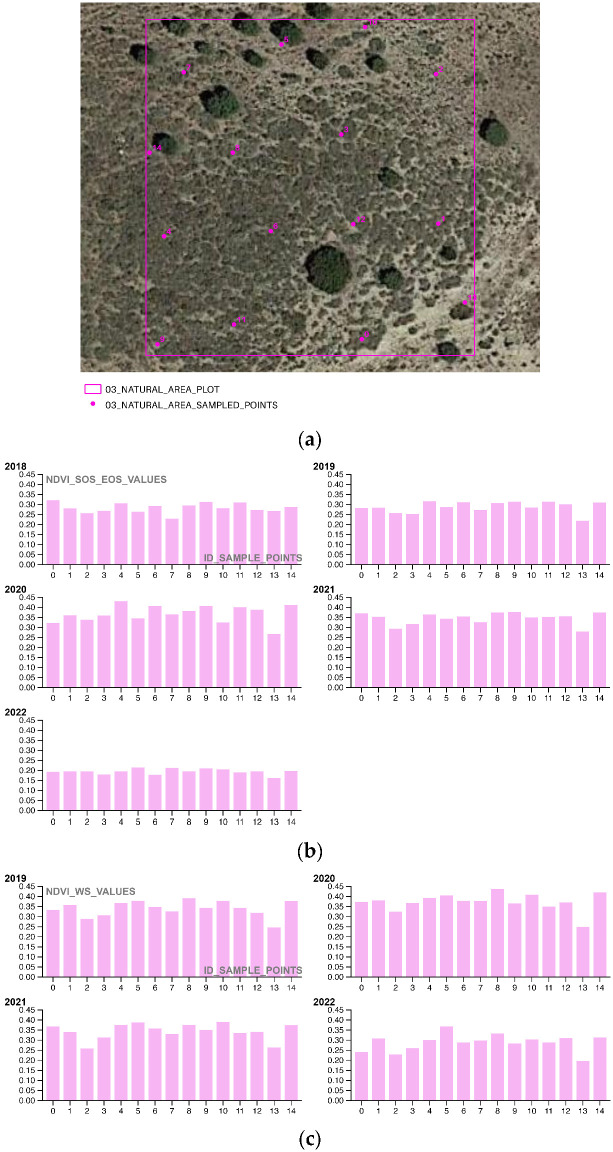
(**a**) 03_NATURAL_AREA_PLOT.shp samples the COPERNICUS/S2_SR NDVI values of a portion in the upper AOI that was reforested with landscape restoration techniques and left to natural management without human interference; (**b**) the COPERNICUS/S2_SR NDVI vegetation trends SOS-EOS phenological growth rate of the sample points (2018–2022) shows the annual trend of vegetation activity during the growing season, from the start of the growing season (SOS) to the end of the growing season (EOS); (**c**) the COPERNICUS/S2_SR NDVI vegetation trends WS phenological growth rate of the sample points (2018–2022) shows the vegetation activity during the winter season (WS).

The third methodological section focuses on analyzing the NDVI SOS-EOS and WS phenological activity of the pioneering restoration actions (Figure 16, Figure 17 and Figure 18) for five successive growing seasons (2018–2022) to evaluate their effectiveness in terms of phenological growth rate and biomass quantification. By annually plotting the sampling values of the 01_REGENERATIVE_ALMONDS_PLOT.shp, 02_CAMP_ALTIPLANO_PLOT.shp, and 03_NATURAL_AREA_PLOT.shp, the study demonstrates that the ERC Camp Altiplano Landscape restoration actions, particularly the forest garden extent that encompasses a portion of the riparian vegetation, are the most effective in promoting vegetation system resilience and reversing land degradation. While all the techniques in place in the AOI are contributing to this cause, the ones implemented in the 02_CAMP_ALTIPLANO_PLOT show the greatest effectiveness in the long-term, even in the WS, when the vegetation is expected to be dormant or less active.

## 5. Discussion

The methods in this research had the primary objective to implement the ERC Soil framework Indicator 16—Above Ground Carbon Capture. This was done with the intention of supporting the establishment of a reference framework for landscape restoration techniques, by taking into consideration the long-term resilience of different ecosystems. The researchers compared the above-ground carbon stocks data based on the satellite-derived reflectance data extracted from both the MODIS/006/MOD13Q1 NDVI and the COPERNICUS/S2_SR NDVI SOS-EOS and WS, 10 m NDVI spatial resolution image. The AOI comprises three areas: 01_Regenerative_Almonds, 02_Altoplano_Camp area, and 03_Natural_Area. Specifically, 01_Regenerative_Almonds belongs to the La Junquera area and is situated in proximity to the 02_Altoplano_Camp area. The management of the almonds is characterized by sustainable production, achieved by the application of new regenerative agricultural techniques also aiming at the regeneration of the depauperated soils. The other area investigated, the 03_Natural_Area above the camp, has a different regeneration approach, as the replantation that occurs is completely managed by natural activity without any human interference or secondary regulation. The research output proved that in the long run, implementing the ERC Landscape restoration techniques (02_Camp_Altiplano) was most successful, demonstrating the effectiveness of these new methodological approaches (described in the Section 3.1.2 and Section 3.1.3) as a valid solution that could be scaled up and thus replicated in the field of ERC ecosystem restoration. To reach this expected final outcome and to retrieve information on the annual land management practices of the three studied portions of the AOI, the researchers exploited the powerful cloud computing of Google Earth Engine (GEE) by calling the GEE API from within R (rGEE) to improve the temporal resolution, spatial resolution, and accuracy of the satellite data. 

### 5.1. Considerations of the Methodological Approach

The methodology consisted of three parts in order to monitor the in-place restoration and regeneration process in the Camp Altiplano area. In the first part, the phenological curve of the high steppe ecosystem in a semi-arid climate was visualized by investigating MODIS/006/MOD13Q1 NDVI for the 20-year time interval from 2000 to 2022. The temporal line chart analysis showed a peak growing season in March–May, with the start of the growing season (SOS) during March and April and the end of the growing season (EOS) in May. It was also observed that the restoration and regeneration actions brought a resilient ecosystem capable of reaching an equilibrium of vegetation during the Winter Season (WS).

The second part involved a more in-depth local-scale analysis of the SOS-EOS and WS values extracted from the COPERNICUS/S2_SR NDVI 10 m spatial resolution image from the year 2017 until 2018–2022, comparing values for the AOI three areas. The plotted boxplot of the SOS-EOS and WS COPERNICUS/S2_SR NDVI showed that the most successful area in the long term was the one implementing the ERC Landscape restoration techniques, which tended to be stable in time (resilience), with a slight decrease in 2022, possibly due to the poor survival rate of the trees and shrubs species planted in the last planting season.

The third part finalized the methodological structurization of the Soil Framework Indicator 16—Above Ground Carbon capture through the extraction of the NDVI values by 15 random generated points, thus, sampling restoration and regeneration actions photosynthetic activity, both in the SOS-EOS and WS periods, by yearly time intervals for the time interval 2018–2022. The studied plots have an area of 20 × 20 square meters and are representative of the different restoration and regeneration techniques implemented in the AOI. This step was crucial to state the fact that there is a tangible benefit in the ERC ecosystem restoration principles and thus finalizes the structurization of the Soil Framework Indicator 16—Above Ground Carbon, opening a new vision for degradation neutrality successful actions scale-up and replication in other ERC Camps of the world. Inizio modulo.

**Fine modulo**

### 5.2. Considerations on the Role of Cloud Computing Platforms (GEE)

The use of cloud computing platforms, such as Google Earth Engine, to process large time-series datasets from the Earth Observation (EO) domain is a powerful tool to monitor above-ground carbon stocks of restoration actions in different environments. This technology enables a better understanding of landscape vegetation changes and assists in the fine-tuning of restoration actions, defining which species and techniques have the best response and performance in drylands, semi-deserts, degraded lands, and other environments. The rGEE methodology can be applied to monitor the ERC Soil Framework Indicator 16-Above Ground Carbon Capture, contributing to achieving the Sustainable Development Goal 15-Life on Land and working towards achieving Land Degradation Neutrality objectives.

The use of GEE cloud computing will offer a new scientific methodological vision for documenting and monitoring regenerative techniques, enabling tracking of tangible impacts in terms of biomass increase in time series. However, it is crucial to validate the vegetation trends derived for each restoration action with on-field registered survival rates of vegetation species and, thus, guide the actions towards a fine-tuning of the chosen reforestation species and management practices. This multiscale holistic approach will allow us to understand which restoration actions perform better in terms of phenological growth rate and guide future policies toward sustainable land management practices. Finally, it is important to correlate this approach with other indicators of the soil reference framework in the specificity of climate, precipitation, and temperature.

### 5.3. Future Integration of the Results into Restor Platform

**Figure 19:**
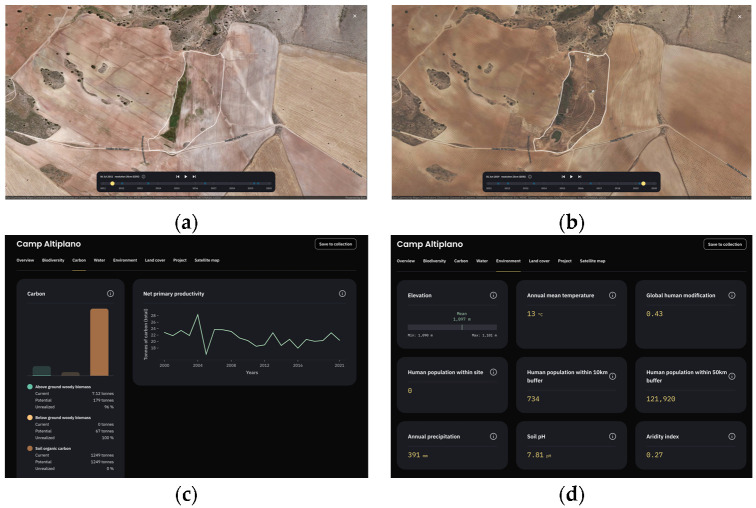
The Restor platform interface, in the specificity of ERC Camp Altiplano site (defined by white boundaries): the timeline before and after restoration (**a**) year 2012 and (**b**) the implemented information in the platform concerning (**c**) carbon and net primary productivity (estimated based on [18]) and (**d**) environmental data monitoring.

The final results and gathered information on the restoration actions of ERC Camp Altiplano’s area will be published on Restor [42] (Figure 19), a platform founded by the Restor Foundation, ETH Zurich, Crowther Lab and developed with Google with the goals of monitoring different types of ecosystems and species present at sites undergoing restoration through remote sensing and variables, and finalizing a predictive model to define how much carbon is accumulating in living biomass. The current methodology to estimate the Net Primary Productivity on the Restor platform is extracted from the MODIS dataset [43] with an average mean value calculated with a 500 m × 500 m (spatial resolution) and an annual aggregation of 8-day composites (temporal resolution).

Sharing the results and the information gathered on the restoration actions of ERC Camp Altiplano’s area on the Restor platform will have several benefits. First, it will allow the local community to access the information and understand the progress made on the restoration project. Second, it will feed the predictive model of the platform, which aims to define how much carbon is accumulating in living biomass and thus contribute to the development of a better predictive model. Third, it will fill the current scale information gap and make it accessible to the Restor community, which will allow for better future scalarization and reproducibility of the project. Fourth, it will provide an opportunity to track the progress made over time and make any necessary adjustments to the restoration project. Overall, sharing the information on the Restor platform will enhance transparency, accountability, and collaboration in the restoration project and contribute to the achievement of the Sustainable Development Goal 15-Life on Land and Land Degradation Neutrality objectives.

## 6. Conclusions 

Implementing a worldwide platform and a defined reference framework (ERC Soil Framework) will enable the scaling up of local efforts to restore environmentally unsustainable degraded and semi-desertified areas towards the global adoption of appropriate mitigation and Land Degradation Neutrality (LDN) policies and actions. This would ideally result in sustainably managed and restored dryland landscapes, which address threats and result in livelihood sustainability, climate change resilience, and adaptiveness.

In order to achieve this goal, it is important to work at multiple scales, engage multiple stakeholders, and employ participatory and user-friendly monitoring techniques to facilitate adaptive and dynamic management in drylands. Local action plans for management should also be integrated to enhance the interaction between people and landscape, promote cultural practices, focus on communities and their activities, and incorporate local knowledge and distinctiveness. Responsibility for future generations and local communities must also be considered in decision-making processes.

Fortunately, environmental monitoring techniques for these studies are relatively simple and cost-effective, allowing for broad application in different geographical contexts where desertification risk is an increasingly important issue. By exploiting the adaptation to climate change by human-driven landscape transformations as powerful drivers of change, the tools available for these studies can help foster continual learning and adaptive management, respond to the dynamic nature of dryland processes, and promote equity.

## Figures and Tables

**Table 1:** Earth Engine MODIS/006/MOD13Q1 select (‘NDVI’)-ImageCollection Metadata, filter Date (‘2002-01-01′, ‘2022-12-01′).

Class	Number of Images	Number of Properties	Number of Pixels *	Approximate Size *
ee$ImageCollection	479	30	41,362,608	11.64 TB

(*) Properties calculated considering a constant geotransform and data type.

**Table 2:** Earth Engine MODIS/006/MOD13Q1 select (‘NDVI’)-Image Metadata (img_index = 0), filter Date (‘2002-01-01′, ‘2022-12-01′).

ID	System: Time_Start	System: Time_End	Number of Bands	Bands Names	Number of Properties	Number of Pixels *	Approximate size *
MODIS/006/MOD13Q1/2002_01_01	2002-01-01	2002-01-17	1	NDVI	6	86,352	24.88 GB

(*) Properties calculated considering a constant geotransform and data type.

**Table 3:** Earth Engine MODIS/006/MOD13Q1 select (‘NDVI’)-Band Metadata (img_band = ‘NDVI’), filter Date (‘2002-01-01′, ‘2022-12-01′).

EPSG (SRID)	Proj4string	Geotransform	Nominal Scale (Meters)	Dimensions	Number ofPixels	Data Type	Approximate size *
MODIS Sinusoidal (SR-ORG:6974)	+proj = sinu + lon_0 = 0 +x_0 = 0 + y_0 = 0 + datum = WGS84 +units = m + no_defs	231,656358263958 0-20015109,353988 0-231,656358263958 10007554,676994	231.6564	86,352	3,350,730	INT	24.88 GB

(*) Properties are calculated considering a constant transform and data type.

**Table 4:** Area of Interest (AOI) Shapefile.

Shapefile	Geometry type	Dimension	Bounding box	Projected CRS
AOI.shp	POLYGON	XY	xmin: 572808,9 ymin: 4201485 xmax: 572869 ymax: 4201546	WGS 84/UTM zone 30N

**Table 5:** Shapefile: 01_Regenerative_Almonds; 02_Camp_Altiplano; 03_Natural_area.

Shapefile	Geometry Type	Dimension	Bounding Box	Projected CRS
01_Regenerative_Almonds.shp	POLYGON	XY	572741,9224055021768436, 4201252,2837546644732356: 572955,4333709812490270, 4201676,1378671433776617	WGS 84/UTM zone 30N
02_Camp_Altiplano.shp	POLYGON	XY	572844,5275248248362914, 4201154,5185987595468760: 573123,6144079214427620, 4201574,1868807338178158	WGS 84/UTM zone 30N
03_Natural_area.shp	POLYGON	XY	572733,6076018153689802, 4201567,7970581464469433: 573152,8252753842389211, 421807,0266833500936627	WGS 84/UTM zone 30N

**Table 6:** Earth Engine COPERNICUS/S2_SR mosaic clipped on (01_Regenerative_Almonds)—ImageCollection Metadata, filter Date (‘2017-03-01′–‘2017-05-31′, SOS-EOS).

Class	Number of Images	Number of Properties	Number of Pixels *	Approximate Size *
ee$ImageCollection	3	23	231,200,370	127.90 GB

(*) Properties calculated considering a constant geotransform and data type.

**Table 7:** Earth Engine COPERNICUS/S2_SR mosaic clipped on (01_Regenerative_Almonds)—Image Metadata (img_index = 0), filter Date (‘2017-03-01′–‘2017-05-31′, SOS-EOS).

ID	System: Time_Start	System: Time_End	Number of Bands	Bands Names	Number of Properties	Number of Pixels *	Approximate Size *
COPERNICUS/S2_SR/20170409T105651_20170409T110529_T30SWH	2017-04-09 11:05:29	2017-04-09 11:05:29	23	B1 B2 B3 B4 B5 B6 B7 B8 B8A B9 B11 B12 AOT WVP SCL TCI_R TCI_G TCI_B MSK_CLDPRB MSK_SNWPRB QA10 QA20 QA60	82	77,066,790	42.63 GB

(*) Properties calculated considering a constant geotransform and data type.

**Table 8:** Earth Engine COPERNICUS/S2_SR mosaic clipped on (01_Regenerative_Almonds)—Band Metadata (img_band = ‘B1′), filter Date (‘2017-03-01′–‘2017-05-31′, SOS-EOS).

EPSG (SRID)	Proj4string	Geotransform	Nominal Scale (Meters)	Dimensions	Number of Pixels	Data type	Approximate size *
WGS 84/UTM zone 30N (EPSG: 32630)	+proj = utm + zone = 30 + datum = WGS84 + units = m + no_defs	60 0 499980 0-60 4300020	60	1831 1830	3,350,730	INT	1.85 GB

(*) Properties calculated considering a constant geotransform and data type.

**Table 9:** Earth Engine COPERNICUS/S2_SR mosaic clipped on (02_Camp_Altiplano)—ImageCollection Metadata, filter Date (‘2021-12-01′,’2022-03-01′, WS).

Class	Number of Images	Number of Properties	Number of Pixels *	Approximate Size *
ee$ImageCollection	14	23	794,911,740	351.49 GB

(*) Properties calculated considering a constant geotransform and data type.

**Table 10:** Earth Engine COPERNICUS/S2_SR mosaic clipped on (02_Camp_Altiplano)—Image Metadata (img_index = 0), filter Date (‘2021-12-01′,’2022-03-01′, WS).

ID	System: Time_Start	System: Time_End	Number of Bands	Bands Names	Number of Properties	Number of Pixels *	Approximate size *
COPERNICUS/S2_SR/20211201T105421_20211201T105655_T30SWH	2021-12-01 11:00:35	2021-12-01 11:00:35	23	B1 B2 B3 B4 B5 B6 B7 B8 B8A B9 B11 B12 AOT WVP SCL TCI_R TCI_G TCI_B MSK_CLDPRB MSK_SNWPRB QA10 QA20 QA60	81	56,779,410	25.11 GB

(*) Properties calculated considering a constant geotransform and data type.

**Table 11:** Earth Engine COPERNICUS/S2_SR mosaic clipped on (02_Camp_Altiplano)–Band Metadata (img_band = ‘B1′), filterDate (‘2021-12-01′,’2022-03-01′, WS).

EPSG (SRID)	Proj4string	Geotransform	Nominal Scale (Meters)	Dimensions	Number of Pixels	Data Type	APPROXIMATE Size *
WGS 84/UTM zone 30N (EPSG: 32630)	+proj = utm + zone = 30 + datum = WGS84 + units = m + no_defs	60 0 499980 0-60 4300020	60	1349 1830	2,468,670	INT	1.09 GB

(*) Properties calculated considering a constant geotransform and data type.

**Table 12:** Earth Engine COPERNICUS/S2_SR SOS-EOS time series filter date.

Year	COPERNICUS/S2_SR SOS Filter Date	COPERNICUS/S2_SR EOS Filter Date
2017	2017-03-01	2017-03-01
2018	2018-03-01	2018-05-31
2019	2019-03-01	2019-05-31
2020	2020-03-01	2020-05-31
2021	2021-03-01	2021-05-31
2022	2022-03-01	2022-05-31

**Table 13:** Earth Engine COPERNICUS/S2_SR WS time series filter date.

Year	COPERNICUS/S2_SR WS start Filter Date	COPERNICUS/S2_SR WS end Filter Date
2019	2018-12-01	2019-03-01
2020	2019-12-01	2020-03-01
2021	2020-12-01	2021-03-01
2022	2021-12-01	2022-03-01

**Table 14:** Polygons of the Sampled areas.

Shapefile	Geometry Type	Dimension	Bounding Box	Projected CRS	Feature Count
01_REGENERATIVE_ALMONDS_PLOT.shp	POLYGON	XY	572801,1651063224999234, 4201413,9108924288302660: 572869,0225870141293854, 4201546,0453444486483932	WGS 84/UTM zone 30N	1
02_CAMP_ALTIPLANO_PLOT.shp	POLYGON	XY	572928,8496651893947273, 4201317,7795310029760003: 573029,3255905595142394, 4201384,1732937581837177	WGS 84/UTM zone 30N	1
03_NATURAL_AREA_PLOT.shp	POLYGON	XY	572929,5905389715917408, 4201609,3690982637926936: 572989,6686835289001465, 4201670,7823126995936036	WGS 84/UTM zone 30N	1

## Data Availability

Not applicable.
